# A Small Membrane Stabilizing Protein Critical to the Pathogenicity of Staphylococcus aureus

**DOI:** 10.1128/IAI.00162-20

**Published:** 2020-08-19

**Authors:** Seána Duggan, Maisem Laabei, Alaa Abdulaziz Alnahari, Eóin C. O’Brien, Keenan A. Lacey, Leann Bacon, Kate Heesom, Chih-Lung Fu, Michael Otto, Eric Skaar, Rachel M. McLoughlin, Ruth C. Massey

**Affiliations:** a School of Cellular and Molecular Medicine, University of Bristol, Bristol, United Kingdom; b Department of Biology and Biochemistry, University of Bath, Bath, United Kingdom; c Host-Pathogen Interactions Group, School of Biochemistry and Immunology, Trinity Biomedical Sciences Institute, Trinity College, Dublin, Ireland; d University of Bristol Proteomics Facility, Biomedical Sciences Building, Bristol, United Kingdom; e Pathogen Molecular Genetics Section, Laboratory of Bacteriology, National Institute of Allergy and Infectious Diseases, U.S. National Institutes of Health, Bethesda, Maryland, USA; f Department of Pathology, Microbiology, and Immunology, Vanderbilt University Medical Center, Nashville, Tennessee, USA; University of Illinois at Chicago

**Keywords:** *Staphylococcus aureus*, cytotoxins, immune evasion, iron homeostasis, virulence

## Abstract

Staphylococcus aureus is a major human pathogen, and the emergence of antibiotic-resistant strains is making all types of S. aureus infections more challenging to treat. With a pressing need to develop alternative control strategies to use alongside or in place of conventional antibiotics, one approach is the targeting of established virulence factors. However, attempts at this have had little success to date, suggesting that we need to better understand how this pathogen causes disease if effective targets are to be identified. To address this, using a functional genomics approach, we have identified a small membrane-bound protein that we have called MspA. Inactivation of this protein results in the loss of the ability of S. aureus to secrete cytolytic toxins, protect itself from several aspects of the human innate immune system, and control its iron homeostasis. These changes appear to be mediated through a change in the stability of the bacterial membrane as a consequence of iron toxicity. These pleiotropic effects on the ability of the pathogen to interact with its host result in significant impairment in the ability of S. aureus to cause infection in both a subcutaneous and sepsis model of infection. Given the scale of the effect the inactivation of MspA causes, it represents a unique and promising target for the development of a novel therapeutic approach.

## Abstract (precis)

Staphylococcus aureus is a major human pathogen, and the emergence of antibiotic-resistant strains is making all types of S. aureus infections more challenging to treat. With a pressing need to develop alternative control strategies to use alongside or in place of conventional antibiotics, one approach is the targeting of established virulence factors. However, attempts at this have had little success to date, suggesting that we need to better understand how this pathogen causes disease if effective targets are to be identified.

## INTRODUCTION

Staphylococcus aureus is a major human pathogen, where the types of infections it causes range in severity from relatively superficial skin and soft-tissue infections (SSTIs) to fatal cases of endocarditis and bacteremia (1, 2). While SSTIs rarely require clinical intervention, more invasive or prolonged infections require antibiotic treatment. Unfortunately, the widespread use of antibiotics has given rise to the emergence of antibiotic-resistant strains of S. aureus, including the notorious methicillin-resistant S. aureus (MRSA), which at its peak was reported to be responsible for in excess of 50% of S. aureus infections in hospitals (3). Infection control measures and changes to antibiotic usage policies have led to a decrease in the incidence of MRSA in several countries. In England, where the surveillance of S. aureus bacteremia is mandatory, the incidence of MRSA declined for several years (by >80% since 2007) (4). However, the incidence of methicillin-sensitive S. aureus (MSSA) bacteremia has increased year on year, having the effect of increasing the overall incidence of S. aureus bacteremia by 30.3% since 2011, when mandatory surveillance began (4), a worrying trend that has also been observed in other countries (5, 6). Although new classes of antibiotics are under development, given the rate at which S. aureus evolves resistance, it is clear that we need to improve our understanding of this pathogen to develop alternative therapeutic strategies.

One approach is to target specific virulence factors through either vaccination or molecular inhibition. To identify and develop such strategies, we need to have a thorough understanding of the pathogenicity of S. aureus, which to date has largely been informed by the analysis of a small number of laboratory strains that have been passaged *in vitro* many times. This work has enabled us to identify and characterize many of the strategies S. aureus uses to cause disease, which, generally speaking, can be split into three categories: adhesion (7), the ability to bind and invade host tissue; toxicity (8), which we describe here as the secretion of proteins that cause host tissue damage; and evasion (9), the ability to neutralize or circumvent the protective activity of the host immune system. Although many specific virulence factors display just one of these three activities, many have multiple activities resulting in them belonging to two and sometimes all three categories, producing a complex picture of how this common member of our normal flora can cause life-threatening disease.

To better understand the pathogenicity of S. aureus, we developed a functional genomics approach to make use of the many thousands of clinical S. aureus isolates that have been sequenced (10–12). This has enabled us to identify novel loci that affect the ability of S. aureus to both secrete cytolytic toxins and form biofilm (10–12). One such gene, which was annotated as encoding a putative membrane-bound protein, MspA, is the focus of this study. Here, we present our characterization of the *in vitro* and *in vivo* properties of the MspA protein, where we have found it to affect many diverse, membrane protein-mediated S. aureus activities. Inactivation of MspA affects toxin production, resistance to innate immune mechanisms, and iron homeostasis, resulting in complete attenuation of this pathogen in both a superficial and invasive model of infection. As such, it represents a promising target for therapeutic development and warrants further investigation.

## RESULTS

### The MspA protein positively affects the production of cytolytic toxins by S. aureus.

**FIG 1:**
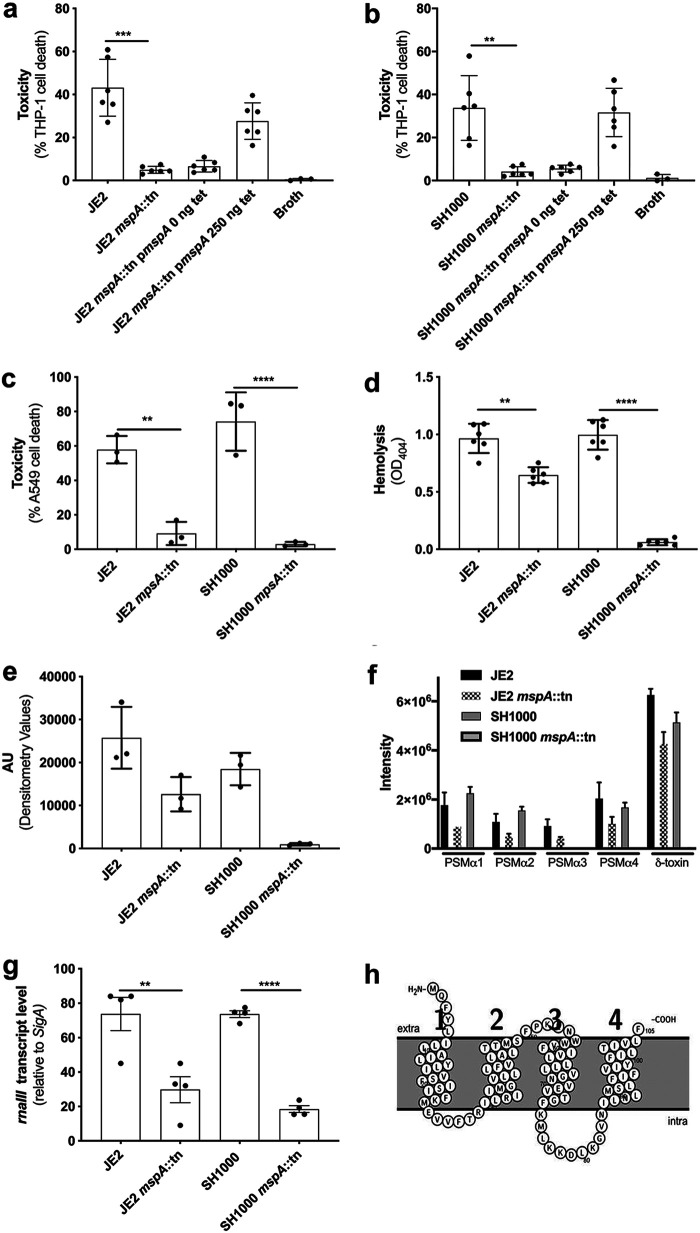
Inactivation of the *mspA* gene results in a loss of toxicity (cytolytic activity) for S. aureus. (a and b) In both the JE2 (a) and SH1000 (b) backgrounds, the inactivation of *mspA* resulted in a loss of cytolytic activity, represented by a significant decrease in cell death. The loss of cytolytic activity was complemented in both backgrounds by expressing the *mspA* gene from a plasmid (p*mspA*). (c and d) Furthermore, the inactivation of *mspA* resulted in a loss of toxicity to a lung epithelial cell line (A549) (c) and human red blood corpuscles (d) in both the JE2 and SH1000 backgrounds. Inactivation of *mspA* also resulted in a reduction in the abundance of alpha toxin in the bacterial supernatants in both backgrounds, determined by Western blotting. (e) Densitometry values for triplicate blots are shown. (f) The effect of the inactivation of *mspA* on secretion of PSMs was quantified by HPLC-MS, where the secretion of delta toxin was the most affected in both backgrounds (*P* < 0.001). AU, arbitrary units. (g) The activity of the Agr system is repressed by the inactivation of *mspA*, as illustrated by qRT-PCR quantification of *rnaIII* transcription. (h) Protter (21) predicts that MspA is a membrane-bound protein with four membrane-spanning domains. The dots represent biological replicates, the bars represent the mean of the replicates, and the error bars are the standard errors of the means. Statistics were performed using a one-way ANOVA, and significance was determined as the following *P* values: *, <0.05; **, 0.01; ***, 0.001; ****, 0.0001.

In previous work, we found an association between a gene with the locus tag SATW20_23930 in the TW20 MRSA background (13) and the ability of clinical strains to lyse human cells (10). We have named the gene *mspA*, for membrane stabilizing protein A, as our data (presented later) suggest it plays a role in the membrane stability of the bacteria. With the availability of a transposon library in the USA300 MRSA background, we sought this gene and found that it was misannotated in the FPR3757 background as intergenic between SAUSA300_2212 and SAUSA300_2213 (14). We were, however, able to obtain transposon mutants in this region from the Nebraska Transposon Mutant Library (NTML) (15), where we found that inactivation of this gene reduced the ability of the bacteria to lyse THP-1 cells, which is an immortalized cell line that is sensitive to the majority of the cytolytic toxins expressed by S. aureus (11, 12) (Fig. 1a). This effect on toxicity was complemented by expressing the *mspA* gene from an inducible promoter on the pRMC2 plasmid (16) (Fig. 1a). To confirm the effect was not specific to this genetic background, we transduced the transposon insertion into a genetically distinct methicillin-sensitive S. aureus (MSSA) strain, SH1000 (17), where again it resulted in the loss of cytolytic activity for the bacteria (Fig. 1b). The effect on toxicity was further confirmed on A549 cells (Fig. 1c) (18) and human red blood corpuscles (Fig. 1d), which contain the additional receptors for S. aureus toxins not present on THP-1 cells, demonstrating the widespread effect the loss of this protein has on cytolytic toxin secretion by both MRSA and MSSA. The effect on toxin production was not due to differences in growth dynamics between wild-type and *mspA* mutants (see Fig. S1 in the supplemental material).

We next quantified the effect the inactivation of MspA has on the expression of several toxins to confirm whether its activity was specific to a single S. aureus toxin or had a more general effect. In both the JE2 and SH1000 background, the inactivation of *mspA* resulted in a decrease in alpha toxin (Hla) secretion, as demonstrated by Western blotting of bacterial supernatant (Fig. 1e and Fig. S2). There is also a reduction in the secretion of several of the phenol-soluble modulins, including delta toxin, which was determined by high-performance liquid chromatography-mass spectrometry (HPLC-MS) (19) (Fig. 1f). For both alpha toxin and the phenol-soluble modulins (PSMs), the effect of the inactivation of *mspA* was more pronounced in the SH1000 background. Given the effect on the production of all these toxins, we hypothesized that the effect of the inactivation of *mspA* was mediated by the repression or lack of activation of the major regulator of toxin expression, the accessory gene regulatory (Agr) quorum-sensing system (20). To test this, we quantified the transcription of the regulatory RNA effector molecule of the Agr system, *rnaIII*, relative to the housekeeping gene *sigA* and found this level to be significantly lower in the *mspA* mutants. This provides a likely explanation for the effect we have observed of the loss of *mspA* on toxicity (Fig. 1g).

To better understand the activity of this protein, we examined both the genomic location of the gene and the likely cellular location of the encoded protein. The *mspA* gene is situated between a hypothetical protein (SAUSA300_2212) and an AcrB/AcrD/AcrF family protein (SAUSA300_2213) (Fig. S3a). To understand the potential cellular localization of the translated protein, we used the protein structure predicting software Protter (21), which suggests that MspA is a membrane-bound protein with four transmembrane domains, both the C and N termini are predicted to be exposed to the outside on the membrane, and it lacks a recognized signal sequence (Fig. 1h). To examine the role of the genes located to either side of the *mspA* gene, we quantified the toxicity of transposon mutants in these genes in both the JE2 USA300 MRSA and SH1000 MSSA backgrounds and found there to be no effect, with the exception of a slight reduction in toxicity for NE42 (SAUSA300_2212) in the JE2 background (Fig. S3b), suggesting that the genes to either side of *mspA* play a minimal role in the effect of MspA on cytolytic toxin production.

### Proteomic analysis reveals pleiotropic effects on the loss of *mspA*.

To identify changes in expression pathways that might explain how the MspA protein is affecting the Agr system, we adopted a proteomic approach and used tandem mass tagging coupled to mass spectroscopy (TMT-MS [22]) on whole-cell lysates of the JE2 strain and its *mspA* mutant. Using the S. aureus USA300 proteome as our reference, we were able to detect and quantify the abundance of 1,149 proteins. Using a 2-fold difference in abundance and a *P* value of less than 0.05 as our cutoff for biological and statistical significance, we found 63 proteins differentially abundant in the *mspA* mutant compared to the wild-type strain (Table 1), demonstrating the pleiotropic effect the loss of *mspA* has on the S. aureus proteome. Three particular differences were of note. The first was that there was a 2.2-fold reduction in the abundance of CrtM (UniProt accession no. Q2FV59), an enzyme involved in the biosynthesis of staphyloxanthin (23, 24). This membrane-bound carotenoid is believed to work alongside the scaffold protein flotillin (FloA) to help form stable functional membrane microdomains (FMMs) (25). While FloA was detected in our analysis, there was no significant difference in its abundance between the wild type and *mspA* mutant. The second observation was that a number of proteins involved in both the uptake (e.g., IsdB and IsdC [26]) and efflux (HrtA and HrtB [27]) of heme-iron were affected; however, there was no detectable difference in the abundance of any other iron uptake or efflux proteins or in the ferric uptake regulatory protein, Fur. Although the extent of the effect on the general response to iron is still unclear, these data suggest that the ability of the bacteria to control iron homeostasis is impaired in the *mspA* mutant. The third difference was a conflicting result where Hla was more abundant in the whole-cell lysate of the mutant; however, we demonstrate above that in the bacterial supernatant Hla is significantly less abundant in the mutant (Fig. 1e), suggesting that protein secretion is affected by the loss of MspA.

**TABLE 1:** Comparative proteomic analysis of whole-cell lysate derived from *mspA* mutant and wild-type strain JE2

Accession no.^*a*^	Gene	Functional description	Fold change^*b*^	Abundance^*c*^	*P* value^*d*^
Q2FVR0	*hrtB*	Hemin transport system permease protein HrtB	18.0	↑	<0.0001
Q2G079	*nrdl*	Protein NrdI	5.8	↑	0.002
Q2G2P2		Globin domain protein	3.6	↑	0.001
Q2FUQ9		Cold shock protein	3.5	↑	0.027
Q2FX98		Uncharacterized protein	3.2	↑	0.003
Q2FZX4	*lipA*	Lipoyl synthase	2.7	↑	<0.0001
Q2FVR1	*hrtA*	Hemin import ATP-binding protein HrtA	2.7	↑	0.006
Q2G0T9		Alpha amylase family protein	2.7	↑	0.003
Q2FXF4		Uncharacterized protein	2.7	↑	0.037
Q2FZA9	*arcC1*	Carbamate kinase 1	2.6	↑	<0.0001
Q8KQR1	*isdC*	Iron-regulated surface determinant protein C	2.6	↑	0.002
P72360	*scdA*	Iron-sulfur cluster repair protein ScdA	2.6	↑	0.006
Q2G1N4		Periplasmic binding protein	2.5	↑	0.008
Q2G1X0	*hla*	Alpha-hemolysin	2.4	↑	0.008
Q2FWY2		Pyrazinamidase/nicotinamidase	2.4	↑	0.029
Q2FV74	*clpL*	ATP-dependent Clp protease ATP-binding subunit	2.4	↑	0.001
Q2G2R5		PTS system lactose-specific IIA component	2.3	↑	0.003
Q2FW75		ABC transporter periplasmic binding protein	2.3	↑	0.002
Q2FVE7	*cntA*	Peptide ABC transporter, peptide-binding protein	2.3	↑	0.004
Q2FWB7		Uncharacterized protein	2.2	↑	0.021
Q2FYK3	*cfvC*	Conserved virulence factor C	2.2	↑	0.004
Q2FXE1		Uncharacterized protein	2.2	↑	0.007
Q2FZB0	*argF*	Ornithine carbamoyltransferase	2.2	↑	<0.0001
Q2FZJ9	*qoxA*	Probable quinol oxidase subunit 2	2.2	↑	0.006
Q2FVF9		Uncharacterized protein	2.1	↑	0.034
Q2FV17	*fda*	Fructose-bisphosphate aldolase class 1	2.1	↑	<0.0001
Q2FZF0	*isdB*	Iron-regulated surface determinant protein B	2.1	↑	0.080
Q2G1Z3		Iron compound ABC transporter	2.1	↑	0.002
Q2FYN6		Uncharacterized hydrolase	2.0	↓	0.155
Q2G0L5	*sdrC*	Serine-aspartate repeat-containing protein C	2.0	↓	0.001
Q2G0W6		Uncharacterized protein	2.1	↓	0.006
Q2FUU5	*lipA*	Lipase 1	2.1	↓	0.002
Q2FWZ8	*ftnA*	Bacterial nonheme ferritin	2.1	↓	0.001
Q2G294		Acetyl-coenzyme A synthetase	2.1	↓	0.001
Q2FZI6	*purH*	Bifunctional purine biosynthesis protein PurH	2.2	↓	<0.0001
Q2FVS2		Uncharacterized protein	2.2	↓	0.001
Q2G2P7	*hutH*	Histidine ammonia-lyase	2.2	↓	0.006
Q2FW51		Truncated major histocompatibility complex class II analog protein	2.2	↓	0.015
Q2FVQ4		l-Lactate permease	2.2	↓	0.006
Q2FV59	*crtM*	Dehydrosqualene synthase	2.2	↓	0.001
Q2FUX7	*arcA*	Arginine deiminase	2.3	↓	0.080
Q2FVE0	*ahpD*	Alkyl hydroperoxide reductase AhpD	2.3	↓	<0.0001
Q2G1C7		Uncharacterized protein	2.3	↓	0.006
Q2FZR3		Oligopeptide ABC transporter	2.3	↓	0.004
Q2FZS2		Truncated major histocompatibility complex class II analog protein	2.4	↓	0.004
Q2G0W8		Uncharacterized protein	2.4	↓	0.008
Q2G2P5	*nikA*	Nickel-binding protein	2.4	↓	0.008
Q2FV34		Uncharacterized protein	2.5	↓	0.001
Q2G118	*parB*	Chromosome partitioning protein, ParB family	2.5	↓	0.005
Q2G1I8		Uncharacterized protein	2.5	↓	0.001
Q2FZS8	*clpB*	Chaperone protein ClpB	2.5	↓	0.001
Q2G087	*hisC*	Histidinol-phosphate aminotransferase	2.6	↓	0.047
Q2G1C9		Uncharacterized protein	2.6	↓	<0.0001
Q2G0G1	*adh*	Alcohol dehydrogenase	2.8	↓	0.017
Q2FWN9		Uncharacterized leukocidin-like protein 2	2.9	↓	<0.0001
Q2G1C8		Uncharacterized protein	3.0	↓	<0.0001
Q2G1D0		Acetyl-coenzyme A acetyltransferase	4.4	↓	0.003
Q2G1K9		Aldehyde-alcohol dehydrogenase	4.6	↓	0.001
Q2FWP0		Uncharacterized leukocidin-like protein 1	4.9	↓	0.003
Q2FVJ5	*bioD*	ATP-dependent dethiobiotin synthetase BioD	6.3	↓	0.001
Q2G218	*ish1*	l-Lactate dehydrogenase 1	7.3	↓	0.001
Q2G091		ABC transporter	9.4	↓	<0.0001
Q2FVJ7	*bioB*	Biotin synthase	9.7	↓	0.001

a Accession number corresponds to the protein identifier in the UniProt database.

b Fold changes of proteins were determined by comparing changes to protein abundances in the JE2*mspA*::tn mutant relative to the level of the wild-type JE2 strain in three independent experiments.

c ↑, Greater abundance in the *mspA* mutant relative to the level in the wild-type strain; ↓, lower abundance in the *mspA* mutant relative to the level in the wild-type strain.

d *P* values were calculated using Student's *t* test.

### MspA mutant is more sensitive to innate immune attack.

**FIG 2:**
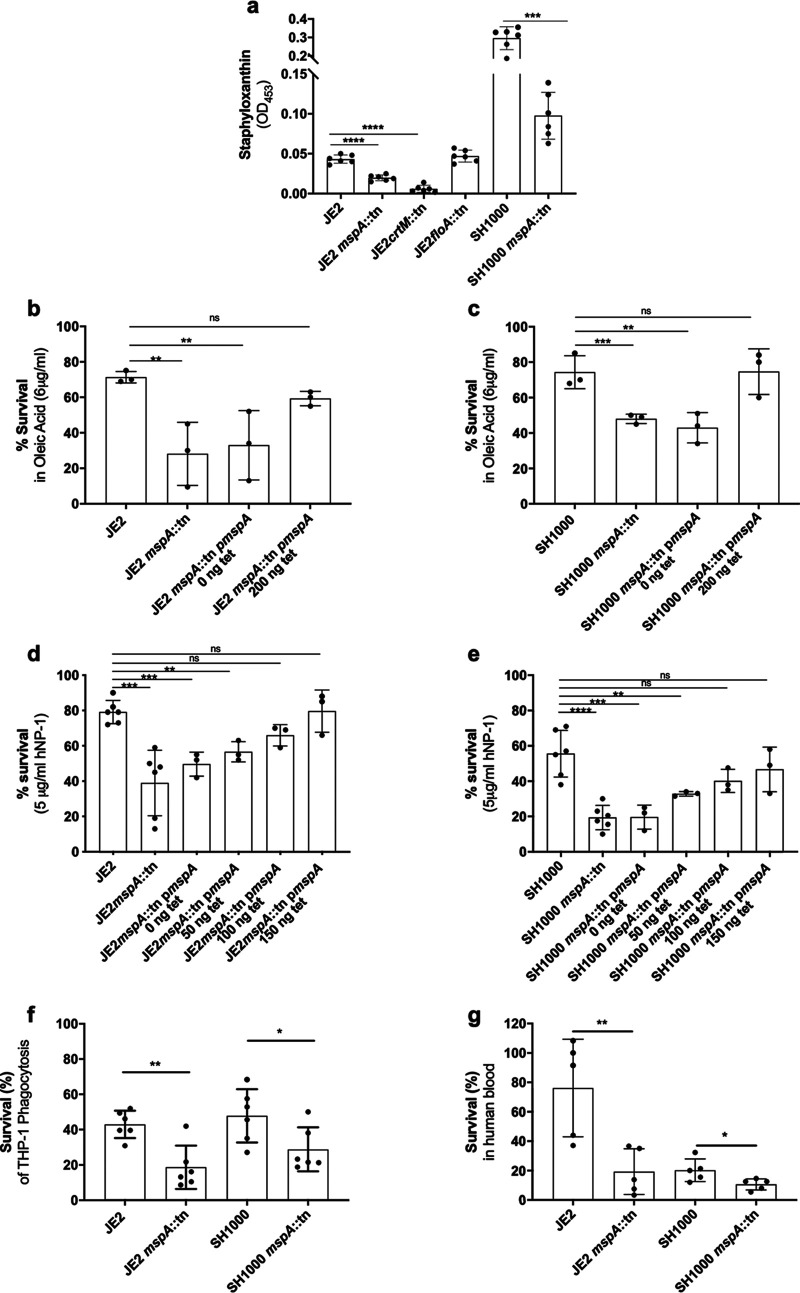
MspA confers protection against aspects of innate immunity. (a) The carotenoid pigment staphyloxanthin is less abundant in the membranes of both the *crtM* and *mspA* mutants than in those of both the wild-type strain and *floA* mutant in the JE2 background. (b and c) Survival of S. aureus upon exposure to oleic acid was reduced in the *mspA* mutants in both S. aureus backgrounds. The survival phenotype was complemented in both backgrounds by expressing the *mspA* gene from a plasmid (p*mspA*). (d and e) Survival of S. aureus upon exposure to the human neutrophil defensin-1 (hNP-1) was reduced in the *mspA* mutants in both S. aureus backgrounds, and this phenotype was complemented in both backgrounds by expressing the *mspA* gene from a plasmid (p*mspA*). (f) Survival of S. aureus following phagocytosis is reduced in the *mspA* mutants in both S. aureus backgrounds. (g) Survival in human blood is reduced in the absence of *mspA* in both backgrounds. The dots represent biological replicates, the bars represent the means from the replicates, and the error bars are the standard errors of the means. Statistics were performed using a one-way ANOVA, and significance was determined as the following *P* values: *, <0.05; **, 0.01; ***, 0.001; ****, 0.0001; ns, not significant.

Staphyloxanthin plays several defensive roles for S. aureus, including protection from reactive oxygen species, fatty acids, and survival inside macrophage, and is believed to work alongside FloA to help form FMMs (23–25). As the proteomic analysis suggested that CrtM (Q2FV59), an enzyme involved in the biosynthesis of staphyloxanthin, was less abundant in the *mspA* mutant, we sought to characterize this effect in greater detail. We first compared the abundance of staphyloxanthin in the membranes of the wild types and *mspA* mutants of both JE2 and SH1000 and in the *crtM* and *floA* mutants in the JE2 background. There was a reduction in both *mspA* mutants; however, they still produced significantly more staphyloxanthin than the *crtM* mutant (Fig. 2a). Given the protective role of staphyloxanthin, we sought to examine whether its reduced abundance in the absence of MspA rendered S. aureus more susceptible to innate immune attack. We first quantified S. aureus resistance to the membrane-damaging effects of human defensin-1 (hNP-1) (28, 29) and oleic acid (30–32). Produced by neutrophils and other immune cells, hNP-1 kills bacteria through pore formation driven by electrostatic attractions between these cationic peptides and the negatively charged bacterial membrane (28, 29). Oleic acid is the predominant bactericidal unsaturated fatty acid naturally present in staphylococcal abscesses and on the skin surface (30–32). Its primary target is also the bacterial membrane, where at high concentrations it increases membrane fluidity and causes protein leakage and interference with metabolic pathways (30–32). The inactivation of *mspA* in both S. aureus backgrounds significantly impaired survival against these innate immune components (Fig. 2b to e). We also investigated bacterial survival inside macrophages (phorbol 12-myristate 13-acetate [PMA]-differentiated THP-1 cells) (Fig. 2f) and following exposure to human blood (Fig. 2g). In both S. aureus backgrounds the *mspA* mutants survived significantly less well than the wild-type strains.

### The loss of MspA affects the stability of the bacterial membrane.

**FIG 3:**
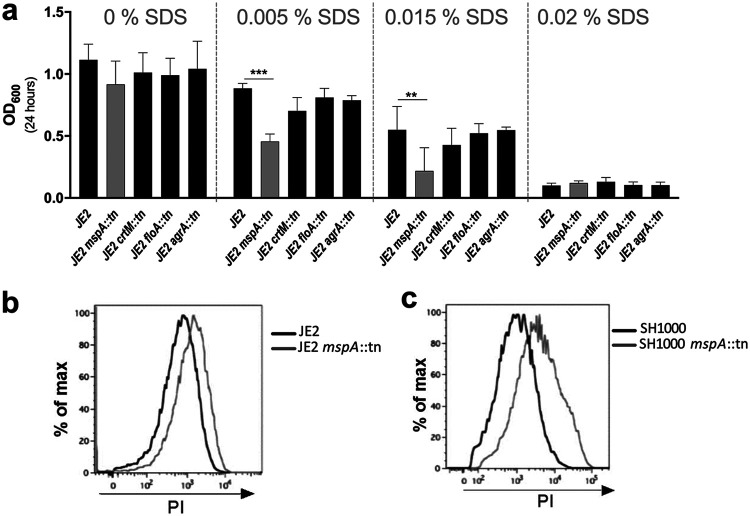
Inactivation of MspA affects membrane stability. (a) Membrane stability was determined by quantifying the sensitivity of the bacteria to the detergent SDS, where the *mspA* mutant was significantly impaired. The sensitivity of mutants with transposons in *crtM*, *floA*, and *agrA* was unaffected and is provided for comparison. Means from three independent biological experiments are shown, and the error bars represent the standard errors of the means. Statistics were performed using a one-way ANOVA, and significance was determined as the following *P* values: *, <0.05; **, 0.01; ***, 0.001. (b and c) The MspA protein inhibits the penetration of the bacterial membrane by propidium iodide. Wild-type and *mspA* mutant cells in the JE2 (b) and SH1000 (c) backgrounds were incubated with PI, and the fluorescence of the cells was analyzed by flow cytometry. The shift of the peak to the right for the *mspA* mutants demonstrates an increased PI signal, indicating that PI was better able to penetrate these cells and stain the DNA. Representative images from three independent experiments are shown.

Our data demonstrate the importance of MspA to the ability of S. aureus to protect itself from the innate immune system, potentially through its effect on staphyloxanthin production. However, that other membrane-associated activities are also affected (i.e., Agr activity) suggests that the loss of MspA has a more general effect on the membrane. To test whether membrane stability was affected by the loss of MspA, we compared the sensitivity of the bacteria to a range of concentrations of the detergent SDS, where we found that the *mspA* mutant was significantly more sensitive to this than the wild-type and *crtM*, *floA*, or *agrA* mutant strains (Fig. 3a). The *mspA* mutants in both backgrounds were also less able to withstand penetration by the DNA-staining dye propidium iodide (Fig. 3b and c), providing further evidence that the loss of MspA causes destabilization of the bacterial membrane. This effect on the membrane may contribute to the reduction in staphyloxanthin in the membrane as well as adding to the sensitization of the membrane to the innate immune system through the reduction of staphyloxanthin in the membrane.

### Iron homeostasis is affected by the loss of MspA.

**FIG 4:**
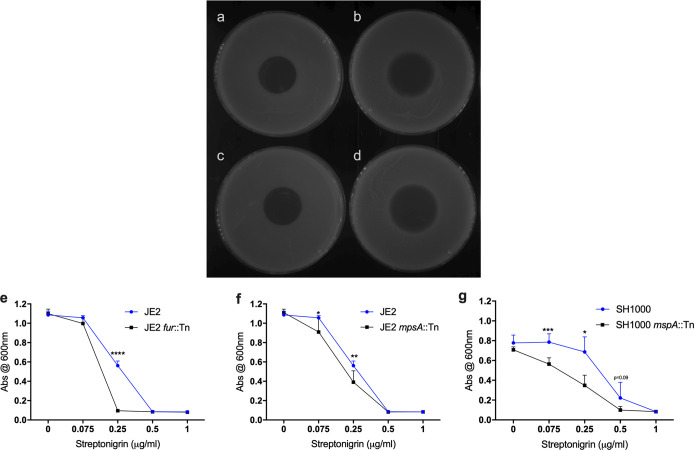
*mspA* mutants contain higher levels of intracellular iron. (a to d) Resistance to streptonigin was used to compare the level of intracellular iron in the *mspA* mutants. Streptonigrin (1.5 μl at a concentration of 2.5 mg/ml) was spotted onto a lawn of JE2 (a), JE2 *mspA::*tn (b), SH1000 (c), and SH1000 *mspA*::tn (d) cells, and the plates were incubated to allow the bacteria to grow. The zone of clearance as a result of the diffusion of the antibiotic was larger for both *mspA* mutants, suggesting they contain higher levels of intracellular iron. Representative images from three independent experiments are shown. (e to g) The effect of streptonigrin was also determined in broth, where bacterial growth was determined after 8 h in increasing concentrations of streptonigrin. A mutant with a transposon in the ferric uptake regulator (*fur*) in the JE2 background was included as a control. Streptonigrin was consistently more effective at inhibiting the growth of the *mspA* mutant strains. Means from six independent biological experiments are shown, and error bars represent the standard errors of the means. Statistics were performed using Student’s paired *t* test, and significance was determined as the following *P* values: *, <0.05; **, 0.01; ***, 0.001; ****, 0.0001.

S. aureus, like many other pathogens, utilizes heme as a source of iron during infection (33). It can either capture hemoglobin and release heme from this or synthesize it endogenously using the enzymes encoded by the *hem* locus (34). However, heme can be toxic at high concentrations, so the balance between uptake, synthesis, and efflux is specifically regulated. The second major observation from the proteomic analysis was that proteins involved in both the uptake and efflux of heme-iron by the bacteria were dysregulated in the *mspA* mutant, suggesting that bacterial iron homeostasis is impaired. To explore this further, we compared the levels of intracellular iron in the wild-type and mutant strains using the antibiotic streptonigrin, which causes nucleic acid damage in the presence of iron (35). As such, quantifying the level of sensitivity of a bacterium to this antibiotic can be used as an indication of the relative amounts of iron present in the bacterial cytoplasm. In both the JE2 and SH1000 backgrounds, the *mspA* mutants had higher levels of intracellular iron, indicated by increased sensitivity to streptonigrin compared to their wild-type strains (Fig. 4a to g).

In the *mspA* mutant, while the increased abundance of the Isd heme uptake system proteins might explain the observed increased levels of intracellular iron, this system is specific to heme, and tryptic soy broth (TSB), the medium used to grow the bacteria, contains negligible amounts of this complex. The Hrt efflux system is also highly specific for heme; that we see increased expression of this suggests that it is responding to the increased levels of heme-iron produced endogenously by the bacteria. However, as its function is to pump heme out of the cells, our seeing an increased iron level despite increased expression of this efflux system suggests that its activity is impaired. Interference in the stoichiometry of ATPases and their permeases has been shown previously to significantly affect the activity of S. aureus efflux systems (36). In the *mspA* mutant, we see an almost 18-fold increased abundance of the HrtB protein (the permease) but only a 2.7-fold increased abundance of the HrtA protein (the ATPase), which is intriguing, given that these genes are cotranscribed. Therefore, the observed differences in relative abundance of the Hrt proteins may affect their efflux activity, which, consequently, would affect the ability of the bacteria to reduce their intracellular heme levels.

**FIG 5:**
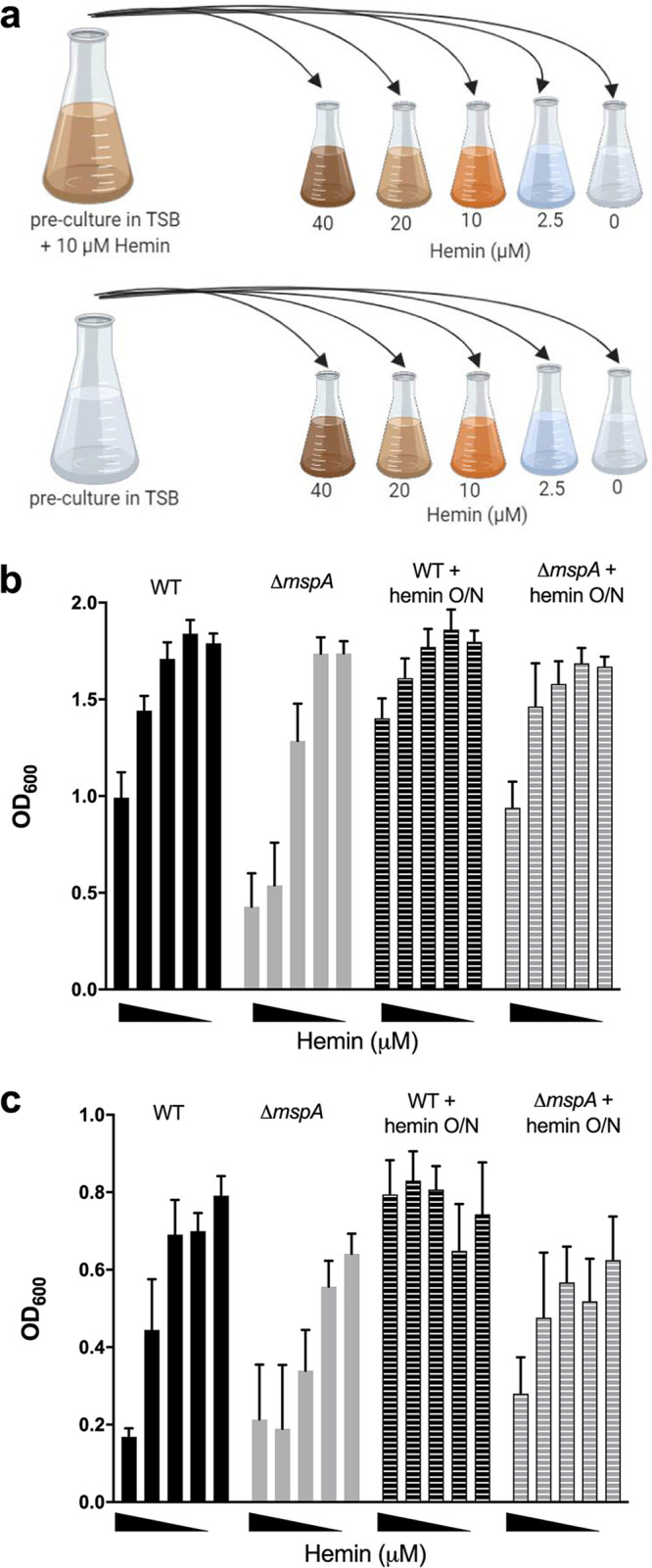
Ability of S. aureus to adapt to hemin is affected by the loss of MspA. (a) Illustration of our hemin adaptation assay. Bacteria were cultured either in normal TSB or in TSB supplemented with 10 μM hemin. Bacteria then were subcultured into a range of hemin concentrations (40, 20, 10, 2.5, and 0 μM). Schematic was prepared on BioRender. (b and c) Preculture in hemin conferred an advantage for subsequent growth in high hemin concentrations in both JE2 (b) and SH1000 (c) backgrounds. O/N, overnight. The loss of *mspA* was disadvantageous for adaption to high hemin environments compared to that of the wild-type strains. Means from six independent biological experiments are shown, and error bars represent the standard errors of the means.

To examine whether the activity of the Hrt system was impaired in the *mspA* mutant despite the HrtA and HrtB proteins being more abundant, we developed a heme adaptation assay. The bacteria were grown overnight in either TSB or TSB supplemented with hemin, and these bacteria then were used to inoculate fresh TSB with increasing concentrations of hemin, where bacterial adaptation to hemin was determined by quantifying their density after 8 h of growth (Fig. 5a). Preexposure of the bacteria to hemin (in the overnight cultures) enabled the wild-type bacteria to adapt to the increasing concentrations of hemin, as illustrated by the higher density of the bacterial cultures after 8 h of growth (for both JE2 and SH1000 wild-type strains at 40 μM, *P* < 0.01) (Fig. 5b and c). However, in both backgrounds, the *mspA* mutants were impaired in their ability to adapt to the presence of hemin relative to the growth of the wild-type strain (for both JE2 and SH1000 backgrounds at 40 μM, *P* < 0.01). This suggests that despite the increased abundance of the Hrt proteins, the efflux activity of this system is impaired, which provides an explanation for the increased intracellular iron concentrations observed (Fig. 4a to f).

### Increased intracellular hemin phenocopies the effects of the loss of MspA.

**FIG 6:**
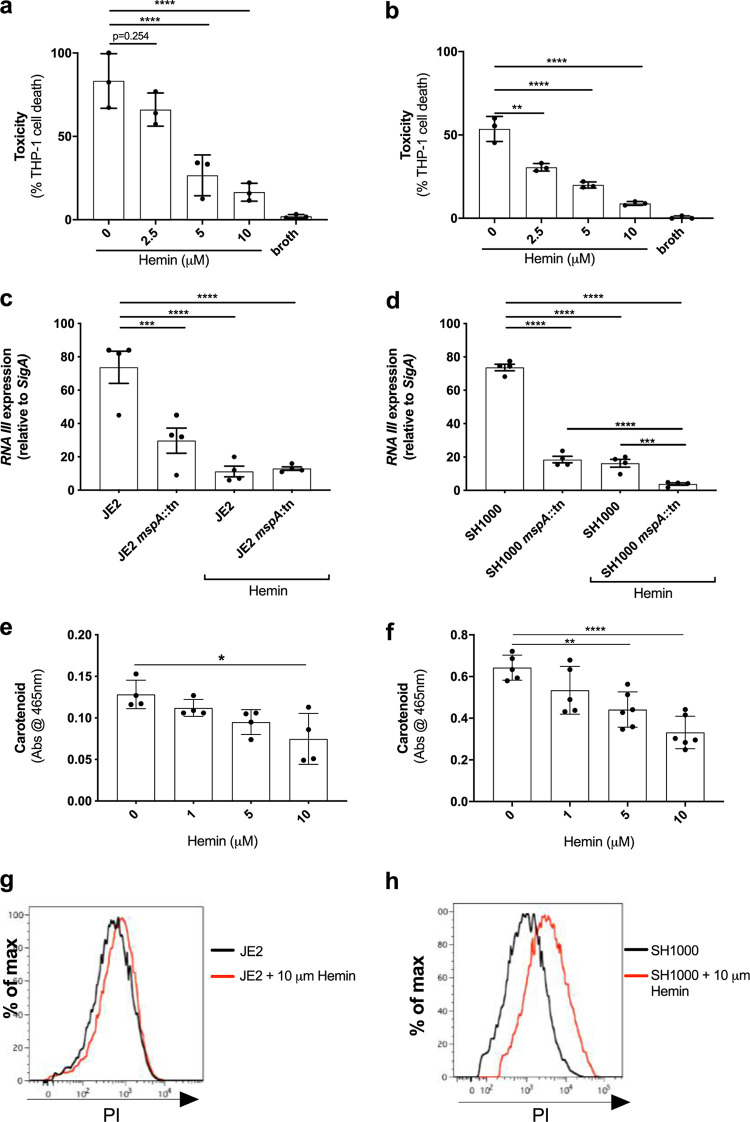
Increased intracellular hemin phenocopies the effects of the loss of MspA. When cultured in increasing concentrations of hemin, the ability of JE2 (a) and SH1000 (b) to cause toxicity in THP-1 cells is diminished in a concentration-dependent manner. (c and d) Transcript levels of RNAIII are, compared to levels of *mspA*-deficient JE2 and SH1000, further reduced in both backgrounds when exposed to hemin. (e and f) Carotenoid biosynthesis is also reduced during culture with hemin, and this effect was observed in a concentration-dependent manner in both backgrounds. (g and h) A shift in the PI staining for JE2 and SH1000 when cultured with hemin suggests that this also affects membrane integrity. Representative images from three independent flow cytometry analyses are shown. The dots represent biological replicates, the bars represent the means from the replicates, and the error bars are the standard errors of the means. Statistics were performed using a one-way ANOVA, and significance was determined as the following *P* values: *, <0.05; **, 0.01; ***, 0.001; ****, 0.0001.

Previous work on the Hrt system, the major iron efflux system of S. aureus, demonstrated that increased intracellular iron can affect what proteins the bacteria secrete (37). As such, we sought to determine whether the increased levels of iron that result from the loss of MspA (Fig. 4) contributes to the loss of toxicity and immune evasion capabilities of the mutants. To address this, we grew the wild-type JE2 and SH1000 strains in TSB with increasing concentrations of hemin, where 10 μM was the highest concentration we could use that did not affect bacterial growth rates. We harvested the bacterial supernatant and found that increasing levels of hemin resulted in decreased cytolytic activity, as measured by THP-1 lysis (Fig. 6a and b). We also verified that, as with the MspA mutant, the effect the increased iron had on toxicity was mediated through the repression or lack of activation of the Agr quorum-sensing system (Fig. 6c and d). Increased levels of intracellular iron also affected the level of staphyloxanthin produced by the bacteria (Fig. 6e and f) and increased the permeability of the bacteria to propidium iodide (Fig. 6g and h). Together, these data suggest that the effect the loss of MspA has on iron homeostasis contributes to the effect we have observed on its offensive and defensive capabilities.

### Without MspA, S. aureus is unable to cause disease in either superficial or invasive murine models of infection.

**FIG 7:**
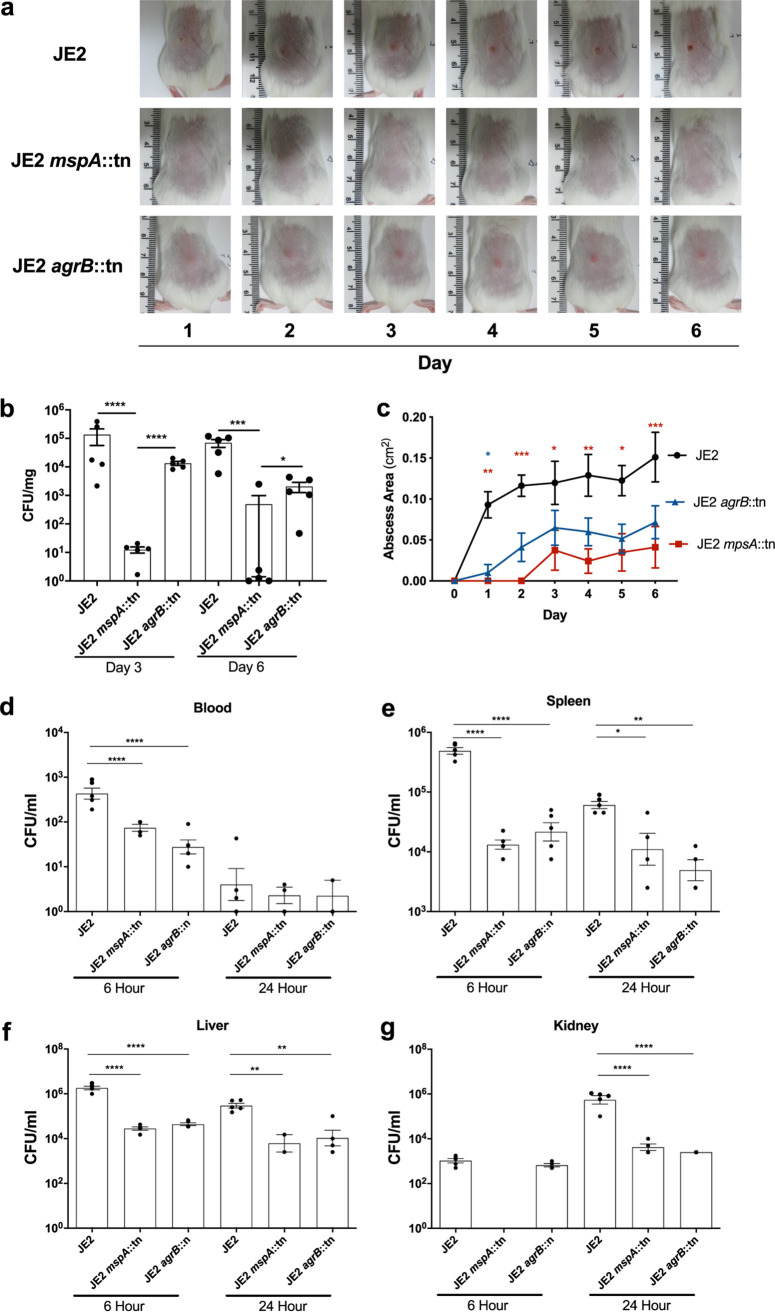
Inactivation of *mspA* affects the ability of S. aureus to cause disease in both a superficial and systemic infection model. BALB/c mice were infected subcutaneously with 2 × 10^7^ CFU wild-type (JE2) and isogenic strains in which the *mspA* and *agrB* genes were inactivated. (a) Abscess lesion area was assessed daily, with representative lesions from the dorsal area of mice from each group shown. (b) The bacterial burden in the skin was assessed by viable counting (CFU/mg) at 3 and 6 days postinfection. (c) Abscess lesion area from panel a expressed as total lesion size (cm^2^) ± SEM. (d to g) Mice were inoculated via tail vein injection of 2 × 10^7^ CFU the wild-type JE2 S. aureus strain and the *mspA* and *agrB* mutants. The tail vein of C57BL/6J mice was inoculated with a sublethal dose of the wild-type JE2 S. aureus. Blood (d), spleens (e), livers (f), and kidneys (g) were harvested at 6 and 24 h postinfection, and the burden of bacteria in each sample was quantified. For the superficial infection, *n* = 10, representative of 2 independent pooled experiments. For the systemic infection, *n* = 5 from one independent experiment. Statistics were performed using a one-way ANOVA with a Tukey posttest (superficial) and Sidak’s multiple-comparison test (systemic), and significance was determined as the following *P* values: *, <0.05; **, 0.01; ***, 0.001; ****, 0.0001.

The loss of the Agr system has been shown in several models of infection to attenuate the pathogenicity of S. aureus (38–40). However, without MspA, S. aureus strains are not only impaired in the activation of the Agr system but also less able to protect themselves from host immunity and to regulate their intracellular iron levels. To examine the effect these pleiotropic effects have *in vivo*, we utilized a murine subcutaneous infection model and compared the *mspA* mutant to both the wild-type JE2 strain and an *agrB* mutant. Photographs of the appearance of the abscesses were captured daily (Fig. 7a), and both the bacterial density in skin punch biopsy specimens (Fig. 7b) and the abscess lesion area (Fig. 7c) were compared for all three strains. As demonstrated previously (38–40), the loss of the Agr system significantly attenuates the ability of S. aureus to cause infection. However, the *mspA* mutant was significantly more attenuated than the *agrB* mutant in terms of both abscess lesion area and tissue bacterial burden in our murine subcutaneous infection model, demonstrating the considerable effect the loss of offensive, defensive, and nutritional capacities has on pathogenicity *in vivo*.

We also compared the pathogenicity of the *mspA* mutant in a murine sepsis model, as although the vast majority of infections caused by S. aureus are superficial skin and soft-tissue infections, invasive diseases, such as sepsis, cause the most concern due to the associated morbidity and mortality. Six and 24 h following sublethal tail vein inoculation of C57BL/6J mice, the density of bacteria in blood, kidneys, and spleen was quantified. In blood after 6 h, both the *agrB* and *mspA* mutants were more effectively cleared than the wild-type strain, and by 24 h all three strains were barely detectable (Fig. 7d). In both the spleens and livers at both 6 h and 24 h postinfection, the wild-type strain was more abundant, and comparable bacterial burdens were detected for the *mspA* and *agrB* mutant strains (Fig. 7e and f). In the kidneys at 6 h postinfection, both the wild type and *agr* mutant were present at similar levels; however, we were unable to detect any *mspA* mutant cells. By 24 h in the kidneys, we were able to detect the *mspA* mutant, and it was at a burden equivalent to that of the *agr* mutant; however, both mutants were at a significantly lower level than the wild-type strain (Fig. 7g). This suggests that both mutants were impaired in their ability to establish an infection in the kidney, where the *mspA* mutant appears to have a greater impairment in its ability to disseminate to the kidneys during the early stages of infection. At this point, we feel an experiment focused specifically on early kidney dissemination is required before any firm conclusions can be drawn from our observations, as this could be due to differences in virulence, tissue tropism, or dissemination as a consequence of any (or all) of the effects the loss of *mspA* has on toxin production, immune evasion, or iron homeostasis.

## DISCUSSION

**FIG 8:**
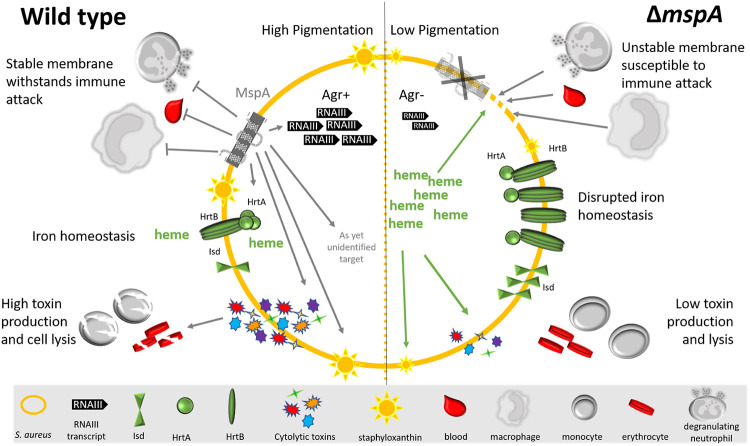
Summary of the effect inactivation of the *mspA* gene has on the bacterial cell. In the absence of MspA, the bacteria are unable to withstand membrane attack and phagocytosis. The Agr system does not become activated, which subsequently affects the production of cytolytic toxins. The abundance and stoichiometry of the Hrt proteins are also affected in the *mspA* mutant, which affects heme-iron homeostasis, and this may contribute to the toxicity and immune susceptibility phenotypes reported here.

S. aureus is a highly adaptable organism that is both a member of the human microbiome, colonizing the nares of up to 30% of healthy populations, and also cause disease in both healthy and immunocompromised individuals (1, 2). If novel means of controlling infection are to be developed, we need a better understanding of its pathogenicity. Through the application of a functional genomics approach to address this, we identified a membrane protein central to the pathogenicity of S. aureus. This protein, MspA, has a relatively small size of 105 amino acids, with only 28 residues predicted to sit outside the bacterial membrane (Fig. 1h). Despite its diminutive status, its loss has pleiotropic effects for which we have created a pictorial summary (Fig. 8). Its loss affects S. aureus offensive (Fig. 1), defensive (Fig. 2 and 3), and nutritional capabilities (Fig. 4 and 5), which together render the bacteria incapable of causing infection (Fig. 7). Although we have yet to uncover the mechanistic detail of how this small protein has such a wide-ranging impact, our data suggest that some of the effects the loss of MspA has are a result of the increased level of heme in the cytoplasm of the *mspA* mutant.

As we continue to study this protein to understand the molecular details of its activity, we have developed two working hypotheses. The first is that MspA directly interacts in some way with the iron homeostasis systems of S. aureus. Our proteomic data showed that the protein subunits of the heme efflux system Hrt were more abundant in the mutant. While increased abundance of a protein is more typically associated with increased activity, here we see the opposite, that the mutant bacteria are less well able to adapt to the toxic effects of hemin. Perhaps MspA physically interacts with these proteins, facilitating their efflux of heme from the cytoplasm. Alternatively, MspA may have a direct structural role in stabilizing the bacterial membrane, such that those proteins that require a certain amount of stability of the membrane cannot function effectively without it. This instability could affect the presentation or accessibility of proteins within the membrane, which might explain why the Hrt and Agr systems are inactive. To test these hypotheses, we are searching for proteins that directly interact with MspA; however, the highly hydrophobic nature of this protein is such that working with any purified forms of it is proving challenging.

National surveillances of S. aureus are reporting year-on-year increases in the incidence of invasive infections (4–6). Given the ability of S. aureus to develop resistance to new antibiotics, alongside our inability to develop an effective vaccine, we need a greater understanding of the fundamental biology of this organism if we are to develop novel therapeutic approaches. Here, we have begun this process, and although the mechanistic detail of MspA activity is not fully established, we highlight the pleiotropic effect its loss has on S. aureus virulence, underlining its potential as a therapeutic target. Encouragingly, recent work by Koch et al. (41) has highlighted the use of small-molecule inhibitors to disrupt membrane protein function, successfully attenuating virulence and reducing mortality in murine models of infection. With further work it is possible that we could identify means of repressing MspA production, which would completely incapacitate the bacteria, rendering it susceptible to immune clearance. As such, this work not only represents a leap in our understanding of a core aspect of the biology of S. aureus but also has identified a potential target for future therapeutic development.

## MATERIALS AND METHODS

### Ethics statement.

Peripheral blood from healthy donors was acquired in accordance with the Declaration of Helsinki and approved by the Research Ethics Committee (REC 18/EE/0265). All animal experiments were conducted in accordance with the recommendations and guidelines of the health product regulatory authority (HPRA), the competent authority in Ireland, and in accordance with protocols approved by Trinity College Dublin Animal Research Ethics Committee.

### Bacterial strains and growth conditions.

A list of S. aureus strains used in this study can be found in Table 2. S. aureus strains were routinely grown in tryptic soy broth (TSB) or brain heart infusion (BHI), where indicated. Overnight cultures were used to inoculate fresh media at a dilution of 1:1,000 and then grown for 18 h at 37°C in air with shaking (180 rpm). For transposon mutants, erythromycin (5 μg/ml) was added to the growth medium. For complementation with pRMC2 plasmid (16) containing the *mspA* gene (p*mspA*), anhydrous tetracycline (50 to 200 ng/ml) was included in the growth medium. The toxin-containing supernatant for each bacterial strain was harvested by centrifugation at 10,000 × *g* for 10 min. Hemin (CAS 16009-13-5) and streptonigrin (CAS 3930-19-6) were included in culture media at the indicated concentrations.

**TABLE 2:** Bacterial strains used in this study

Strain	Description	Reference
JE2	USA300; community-acquired MRSA, type IV SCC*mec*; lacking plasmids p01 and p03; wild-type strain of the NTML	15
JE2 *agrB*::tn	Accessory gene regulator B (*agrB*) transposon mutant in JE2	15
JE2 *mspA*::tn	*mspA* transposon mutant in JE2	15
JE2 *mspA*::tn p*mspA*	*mspA* transposon mutant complemented with *mspA* gene housed in pRMC2 expression plasmid	This study
SH1000	Laboratory strain, 8325-4 with a repaired *rsbU* gene; SigB positive	17
SH1000 *mspA*::tn	*mspA* transposon mutant in SH1000	This study
SH1000 *mspA*::tn p*mspA*	*mspA* transposon mutant complemented with *mspA* gene housed in pRMC2 expression plasmid	This study
JE2 *crtM*::tn	Dehydrosqualene desaturase mutant in JE2	15
JE2 *floA*::tn	Flotillin mutant in JE2	15
JE2 *fur*::tn	Ferric uptake regulator mutant in JE2	15

### Genetic manipulations involving *mspA*.

The *mspA* gene was amplified by PCR from JE2 using Phusion high-fidelity DNA polymerase (NEB) and primers MspFW, CGGGTACCGAACCCTTTGAAACG (KpnI is underlined; melting temperature [*T_m_*], 64.2°C), and MspRV, GCGAGCTCGTTGCAATTATGTTATTGC (SacI is underlined; *T_m_*, 63.4°C), and cloned into the tetracycline-inducible plasmid pRMC2 to make p*mspA*. This was electroporated into S. aureus RN4220 and subsequently into JE2 to complement the *mspA* transposon mutant. DNA from JE2*mspA*::Tn was transduced into wild-type SH1000 by transduction with Φ11, and transductants containing the inserted transposon were screened on tryptic soy agar (TSA) containing erythromycin (10 μg/ml). SH1000*mspA*::Tn was verified for Tn insertion of *mspA* by colony PCR using the above-described *mspA* primers.

### Monocyte (THP-1) toxicity.

The monocytic THP-1 cell line (ATCC TIB-202) was used as previously described (8, 9). Briefly, cells were grown in 30 ml of RPMI 1640 supplemented with heat-inactivated fetal bovine serum (10%), l-glutamine (1 μM), penicillin (200 U/ml), and streptomycin (0.1 mg/ml) (defined as complete medium) in a humidified incubator at 37°C with 5% CO_2_. For toxicity assays, cells were harvested by centrifugation at 400 × *g* and resuspended to a final density of 1 × 10^6^ to 1.5 × 10^6^ cells/ml in tissue-grade phosphate-buffered saline (PBS), typically yielding >95% viability. These were then incubated for 10 min in the harvested supernatant of bacteria grown for 16 h in TSB (at a dilution of 30% for the SH1000 strains and at 10% for the JE2 strains), and THP-1 cell death was quantified by trypan blue exclusion and easyCyte flow cytometry. Experiments were performed in triplicate three times, and results represent the means ± standard deviations (SD).

### Bronchial epithelial cell (A549) toxicity.

A549 cells were grown in complete media. When confluent (80 to 90%), cells were detached with trypsin EDTA (0.25%; ThermoFisher), resuspended, centrifuged for 10 min at 400 × *g*, and resuspended to 1 × 10^6^ to 1.5 × 10^6^ cells/ml in tissue-grade PBS. To determine S. aureus toxicity, 75 μl of bacterial supernatant (bacterial grown overnight at 37°C in TSB) diluted to 25% with fresh broth was incubated with 75 μl of A549 cells in a 96-well plate for 20 min at 37°C. Cell lysis was measured as lactate dehydrogenase release using the CytoTox 96 nonradioactive cytotoxicity assay (Promega) according to the manufacturer’s instructions. Experiments were performed in triplicate three times, and results represent the means ± SD.

### Human red blood corpuscle toxicity.

Human red blood corpuscles (RBCs) were isolated from heparinized venous blood obtained from healthy adult volunteers. RBCs were washed twice in sterile saline (0.9% NaCl) and centrifuged at 600 × *g* for 10 min. RBCs were diluted to 1% in PBS, and 200 μl was incubated with 50 μl of bacterial supernatant in a 96-well plate for 30 min at 37°C. Plates were centrifuged for 5 min at 400 × *g*, supernatants were transferred to a sterile 96-well plate, and RBC lysis was evaluated by determining the absorbance at 404 nm. Saline and 0.5% Triton X-100 were used as negative and positive controls, respectively.

### Toxin expression quantification.

For Hla quantification, overnight cultures of S. aureus were diluted 1:1,000 in 5 ml TSB and incubated for 18 h at 37°C with shaking (180 rpm). Bacteria were normalized to an optical density at 600 nm (OD_600_) of 2 and centrifuged for 10 min at 10,000 × *g*, supernatant was removed, and proteins were precipitated using trichloroacetic acid (TCA) at a 20% final concentration for 2 h on ice. Samples were centrifuged at 18,000 × *g* for 20 min at 4°C, washed three times in ice-cold acetone, and solubilized in 100 μl 8 M urea. Proteins (10 μl of each sample) were mixed with 2× concentrated sample buffer and heated at 95°C for 5 min before being subjected to 12% SDS-PAGE. Separated proteins were wet transferred onto a nitrocellulose membrane and afterwards blocked overnight in 5% semiskim milk at 4°C. Membranes were washed and incubated with polyclonal antibodies specific for alpha-toxin (1:5,000 dilution; Sigma-Aldrich) for 2 h at room temperature. Membranes were washed and incubated with horseradish peroxidase-coupled protein G (1:1,000; Invitrogen) for 1 h at room temperature. Proteins were detected by using the Opti-4CN detection kit (Bio-Rad). The Western blot analyses were performed in triplicate, and the bands were scanned and quantified by using ImageJ software (http://rsbweb.nih.gov/ij/). Phenol-soluble modulin (PSM) quantification, including delta toxin, was measured using reverse-phase high-performance liquid chromatography/mass spectrometry (RP-HPLC/MS) as described previously (19).

### Quantitative reverse transcription-PCR (qRT-PCR).

Cultures of S. aureus grown overnight in TSB were diluted 1:1,000 in fresh TSB and grown at 37°C for 7 h. Cultures were normalized based on OD_600_ measurements prior to RNA isolation. Cultures were treated with two volumes of RNAprotect (Qiagen), incubated for 10 min at room temperature, and centrifuged, and the pellet was resuspended in Tris-EDTA (TE) buffer (Ambion) with lysostaphin (5 mg/ml) and incubated for 1 h, followed by proteinase K treatment for 30 min. RNA was isolated using the Quick-RNA kit (Zymo Research). RNA was quantified using a NanoDrop (Thermo Fisher Scientific). Reverse transcription was performed using the qScript cDNA synthesis kit QuantaBio) according to the manufacturer’s instructions using random primers. Standard curves were generated for both *sigA* (forward, 5′-AACTGAATCCAAGTCATCTTAGTC-3′; reverse, 5′-TCATCACCTTGTTCAATACGTTTG-3′) and *rnaIII* (forward, 5′-GAAGGAGTGATTTCAATGGCACAAG-3′; reverse, 5′-TCATCACCTTGTTCAATACGTTTG-3′) primers using genomic DNA to determine efficiency. Real-time PCR was performed using the SYBR green qPCR mix (NeoBiotech) and the Mic qPCR Cycler (Bio Molecular Systems). Cycling conditions were 95°C for 10 min, followed by 40 cycles of 95°C for 15 s and 60°C for 1 min and a dissociation step at 95°C for 15 s and 60°C for 1 min. Cycle threshold (*C_T_*) values were determined for at least 3 biological repeats. For each reaction, the ratio of RNA III and *sigA* transcript number was calculated as 2^(^*^CT^* ^RNAIII –^ *^CT sigA^*^)^.

### Protein extraction, TMT labeling, and high-pH reverse-phase chromatography.

Bacteria were cultured for 18 h in TSB, and 1 ml of this was pelleted by centrifugation and washed three times with PBS. The pellet was resuspended in 200 μl of PBS containing lysostaphin (200 μg/ml), DNase I (20 μg/ml), and RNase (10 μg/ml) and incubated at 37°C for 1 h. Insoluble debris was removed by centrifugation, and the samples were adjusted with PBS to a concentration of 2 mg/ml. Aliquots of 100 μg (50 μl) of the samples then were digested with trypsin (2.5 μg trypsin per 100 μg protein; 37°C, overnight) and labeled with TMT 10-plex reagents according to the manufacturer’s protocol (Thermo Fisher Scientific), and the labeled samples were pooled. Each sample being differentially labeled facilitates the analysis of multiple samples in parallel. To identify and quantify each protein within the pooled labeled samples, an aliquot was evaporated to dryness and resuspended in buffer A (20 mM ammonium hydroxide, pH 10) prior to fractionation by high-pH reverse-phase chromatography using an Ultimate 3000 liquid chromatography system (Thermo Fisher Scientific). In brief, the sample was loaded onto an XBridge BEH C_18_ column (130 Å, 3.5 μm, 2.1 mm by 150 mm; Waters, UK) in buffer A, and peptides were eluted with an increasing gradient of buffer B (20 mM ammonium hydroxide in acetonitrile, pH 10) from 0% to 95% over 60 min. The resulting fractions were evaporated to dryness and resuspended in 1% formic acid prior to analysis by nano-LC MS/MS using an Orbitrap Fusion Tribrid mass spectrometer (Thermo Scientific).

### Nano-LC mass spectrometry.

High-pH RP fractions were further fractionated using an Ultimate 3000 nanoHPLC system in line with an Orbitrap Fusion Tribrid mass spectrometer (Thermo Scientific). In brief, peptides in 1% (vol/vol) formic acid were injected onto an Acclaim PepMap C_18_ nanotrap column (Thermo Scientific). After washing with 0.5% (vol/vol) acetonitrile, 0.1% (vol/vol) formic acid, peptides were resolved on a 250-mm by 75-μm Acclaim PepMap C_18_ reverse-phase analytical column (Thermo Scientific) over a 150-min organic gradient, using seven gradient segments (1 to 6% solvent B over 1 min, 6 to 15% B over 58 min, 15 to 32% B over 58 min, 32 to 40% B over 5 min, 40 to 90% B over 1 min, held at 90% B for 6 min and then reduced to 1% B over 1 min) at a flow rate of 300 nl min^− 1^. Solvent A was 0.1% formic acid, and solvent B was aqueous 80% acetonitrile in 0.1% formic acid. Peptides were ionized by nano-electrospray ionization at 2.0 kV using a stainless steel emitter with an internal diameter of 30 μm (Thermo Scientific) and a capillary temperature of 275°C.

All spectra were acquired using an Orbitrap Fusion Tribrid mass spectrometer controlled by Xcalibur 2.0 software (Thermo Scientific) and operated in data-dependent acquisition mode using an SPS-MS3 workflow. FTMS1 spectra were collected at a resolution of 120,000 with an automatic gain control (AGC) target of 200,000 and a maximum injection time of 50 ms. Precursors were filtered with an intensity threshold of 5,000 according to charge state (to include charge states 2 to 7) and with monoisotopic precursor selection. Previously interrogated precursors were excluded using a dynamic window (60 s ± 10 ppm). The MS2 precursors were isolated with a quadrupole mass filter set to a width of 1.2 *m/z*. ITMS2 spectra were collected with an AGC target of 10,000, maximum injection time of 70 ms, and collision-induced dissociation (CID) collision energy of 35%.

For FTMS3 analysis, the Orbitrap was operated at 50,000 resolution with an AGC target of 50,000 and a maximum injection time of 105 ms. Precursors were fragmented by high-energy collision dissociation (HCD) at a normalized collision energy of 60% to ensure maximal TMT reporter ion yield. Synchronous precursor selection (SPS) was enabled to include up to five MS2 fragment ions in the FTMS3 scan.

### Proteomic data analysis.

The raw data files were processed and quantified using Proteome Discoverer software v2.1 (Thermo Scientific) and searched against the UniProt Staphylococcus aureus strain USA300 database (https://www.uniprot.org/taxonomy/451516) using the SEQUEST algorithm (42). Peptide precursor mass tolerance was set at 10 ppm, and MS/MS tolerance was set at 0.6 Da. Search criteria included oxidation of methionine (+15.9949) as a variable modification and carbamidomethylation of cysteine (+57.0214), the addition of the TMT mass tag (+ 229.163) to peptide N termini, and lysine as fixed modifications. Searches were performed with full tryptic digestion, and a maximum of two missed cleavages was allowed. The reverse database search option was enabled, and all peptide data were filtered to satisfy a false discovery rate of 5%.

### Carotenoid pigment analysis.

Carotenoid pigment analysis was performed as described previously (43), with minor modifications. Overnight bacterial cultures were used to inoculate 2 ml of fresh TSB in a 1:1,000 dilution, which was subsequently grown for 24 h at 37°C with shaking (180 rpm). Eight hundred fifty microliters of bacterial culture was centrifuged at 10,000 × *g* for 4 min, supernatant was discarded, and cells were resuspended in 100% methanol. Cells were heated for 3 min at 55°C in a water bath and centrifuged at 10,000 × *g* for 2 min to remove cell debris, and extraction was repeated twice. The absorbance of the methanol extracts was measured at 453 nm using a photometer (GeneSpec III; Hitachi). JE2 *crtM*::Tn was used as a negative control.

### Oleic acid susceptibility.

Bacteria were grown overnight, subcultured 1:1,000 in fresh TSB, and grown for 18 h. Bacteria were washed twice in 2 M NaCl–2mM EDTA buffer, normalized to an OD_600_ of 1, and further diluted 1:1,000 in this buffer. Oleic acid (Sigma) was initially dissolved in ethanol, and a working solution was further prepared in 2 M NaCl-2mM EDTA buffer. One hundred microliters of cells was incubated with either 100 μl of buffer or oleic acid solution (6 μg/ml final concentration) in duplicate for 1 h at 37°C. Bacteria were enumerated following dilution in PBS and plating onto TSA. Bacterial survival was calculated by dividing the number of bacteria from wells containing oleic acid by bacteria from wells containing the control.

### Antimicrobial peptide susceptibility.

The human neutrophil defensin-1 (hNP-1) (AnaSpec Incorporated, CA, USA) susceptibility assay was performed as described previously (15). Briefly, a final inoculum of 10^5^ CFU was resuspended in 1% BHI supplemented with 10 mM potassium phosphate buffer and a final concentration of 5 μg/ml of hNP-1 and incubated for 2 h at 37°C. Final bacterial concentration was evaluated by serial plating onto TSA plates, and data are represented as mean (±SD) percent survival of CFU.

### Streptonigrin agar and broth susceptibility assay.

For the agar assay normalized OD_600_, overnight cultures were diluted 1:100 in PBS, and 100 μl was mixed with 3 ml 0.5% agar and poured over TSA plates. When dry, 1.5 μl of streptonigrin (2.5 mg/ml; dissolved in dimethyl sulfoxide) was spotted onto the plate. Plates were incubated at 37°C for 18 h, and zones of clearance were measured. Data are represented as areas of growth inhibition. For the broth assays, normalized overnight cultures were diluted 1:1,000, and 10 μl of this was used to inoculate 5 ml of TSB containing increasing concentrations of streptonigrin (0, 0.075, 0.25, 0.5, and 1 μg/ml). The relative sensitivity of the bacteria to streptonigrin, as a proxy for intracellular iron levels, was determined by quantifying bacterial growth (OD_600_) after 8 h of incubation at 37°C.

### Phagocytosis assay.

THP-1 cells were harvested by centrifugation and resuspended in fresh complete medium to 2 × 10^5^ cells/ml. Monocytes were differentiated by the addition of phorbol 12-myristate 13-acetate (PMA) at a final concentration of 100 nM, and 500 μl of cells was added to a tissue culture-treated 24-well plate (Nunc) for 48 h. THP-1 cells were washed twice in tissue-grade PBS and incubated with complete medium 24 h before infection. Two hours before infection, THP-1 cells were washed twice in PBS and incubated in complete medium without antibiotics. Bacterial strains were grown overnight, diluted 1:100 in fresh TSB, and grown to an OD of 0.3. Bacterial cells were washed twice in PBS, and a multiplicity of infection of 1 was established to infect THP-1 cells. Plates were centrifuged at 300 × *g* for 5 min to synchronize phagocytosis and incubated at 37°C with 5% CO_2_ for 1 h. Following 1 h of incubation, medium was discarded, cells were washed four times in PBS, and wells were incubated with RPMI medium containing gentamicin (200 μg/ml) and lystostaphin (20 μg/ml) for 1 h. Medium was discarded, wells for 2-h time points were lysed with Triton X-100 (0.01%) and CFU enumerated on TSA plates, and wells for analysis at 6 h were further incubated in RPMI containing no antibiotics and processed as described above.

### Human blood survival assay.

Blood was collected from healthy volunteers and anticoagulated with citrate. Bacterial cells were cultured to stationary phase for 18 h, diluted 1:100 in fresh TSB, and grown to an OD_600_ of 0.6. Bacteria were washed in PBS three times, and 1 × 10^6^ cells were inoculated into 1 ml blood and incubated at 37°C for 60 min with gentle rotation. Serial dilutions were plated on TSA to determine the number of CFU. The same number of bacterial cells inoculated into PBS and diluted and plated immediately acted as a control. Survival was determined as the percentage of CFU in blood relative to the PBS control.

### SDS stability.

The S. aureus strains were grown overnight in TSB and used at a 1:1,000 dilution to inoculate TSB containing a range of concentrations of sodium dodecyl sulfate (Sigma). The ability of the bacteria to withstand the membrane-damaging effect of the detergents was determined by quantifying bacterial growth (OD_600_) after 24 h using a SPECTROstar Nano plate reader (BMG Labtech).

### PI permeability assay.

Membrane stability was determined as previously described (44, 45). The S. aureus strains were grown overnight in TSB. Cells were harvested by centrifugation and resuspended in PBS to a concentration of 1 × 10^6^ cells per ml. One hundred microliters of bacterial cells was incubated with propidium iodide (PI) for 5 min at room temperature prior to flow cytometric analysis on a Novocyte (ACEA Biosciences). Data were analyzed using FlowJo 10.5.

### Mice.

Age (6 to 8 weeks)- and sex-matched wild-type BALB/c and C57BL/6J mice were purchased from Charles River Laboratories UK. Mice were housed under specific-pathogen-free conditions at the Trinity College Dublin Comparative Medicines unit. All animal experiments were conducted in accordance with the recommendations and guidelines of the health product regulatory authority (HPRA), the competent authority in Ireland, and in accordance with protocols approved by Trinity College Dublin Animal Research Ethics Committee.

### Murine subcutaneous abscess model.

The dorsal backs of mice were shaved and injected subcutaneously with S. aureus (2 × 10^7^ CFU) in 100 μl of sterile PBS using a 27-gauge syringe (BD Biosciences). Measurements of abscess lesion area (in square centimeters) were made by analyzing digital photographs using M3 Vision software (Biospace Lab), and pictures contain a millimeter ruler as a reference. To determine the bacterial burden, 8-mm punch biopsy specimens of lesional skin were taken at days 3 and 6 postinfection. Tissue was homogenized in sterile PBS, and total bacterial burden was determined by plating out serial dilutions on TSA.

### Murine bloodstream infection model.

A total of 5 × 10^7^ cells of the JE2, JE2*mspA*::tn, or JE2*agrB*::tn strain was used via tail vein injection. Mice were culled at 6 and 24 h. Blood was collected by cardiac puncture. Liver, spleen, and kidney were harvested and homogenized in 1 ml of PBS. Bacterial burdens were established by plating out serial dilutions of blood and organ homogenates on TSA.

### Statistics.

Paired two-tailed Student's *t* test or one-way analysis of variance (ANOVA) (GraphPad Prism v5.0) were used to analyze the observed differences between experimental results. A *P* value of <0.05 was considered statistically significant. For *in vivo* studies, two-way ANOVA with Tukey posttest or Sidak’s multiple-comparison test were used to analyze differences between groups.

## Supplementary Material

Supplemental file 1
