# Supplementary material for: A Small Membrane Stabilizing Protein Critical to the Pathogenicity of Staphylococcus aureus
Source: Infect Immun. 2020 Aug 19;88(9):e00162-20. doi: 10.1128/IAI.00162-20 (PMC7440758; doi:10.1128/IAI.00162-20)
Supplement: Supplemental file 1 [file IAI.00162-20-s0001.pdf]

Supplementary Data

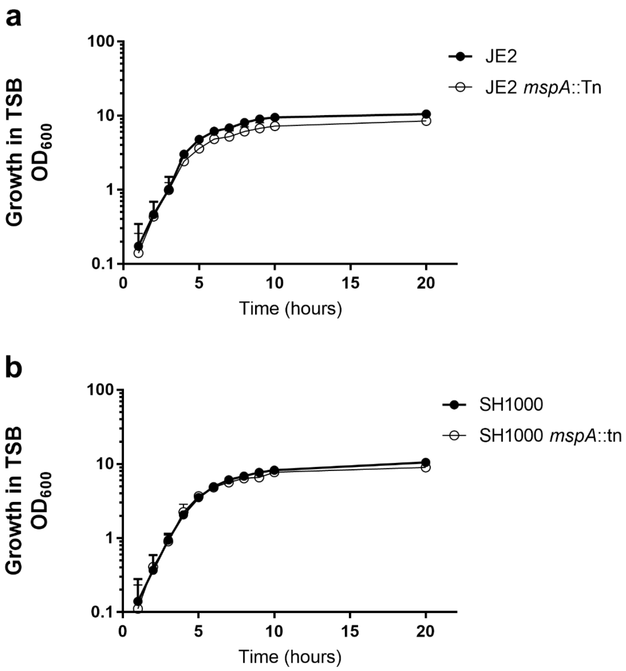

Figure S1: Growth curve analysis of (a) JE2 and JE2 *mspA*::tn and (b) SH1000 and SH1000 *mspA*::tn in TSB. Data shown are the means of three biological replicates.

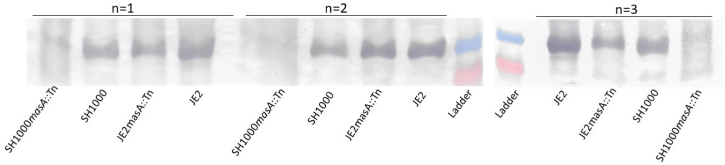

Figure S2: The effect of *mspA* deletion on alpha toxin secretion was determined by Western blotting using anti-alpha toxin antibodies. These blots were scanned, the relative quantity of alpha toxin quantified using ImageJ and these data presented in Figure 1E). Blots from three independent experiments are shown.

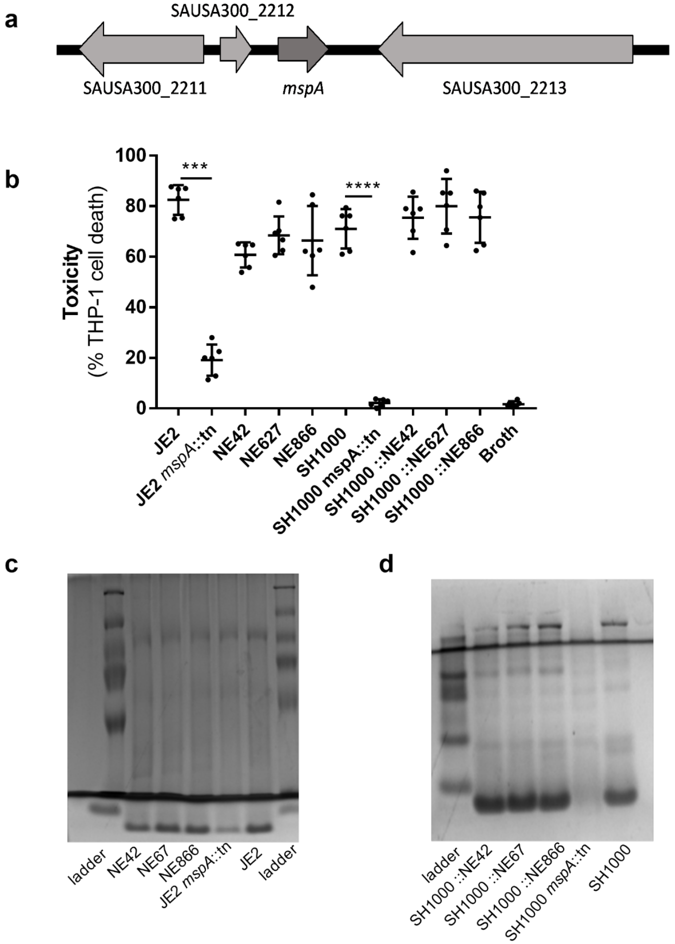

Figure S3: (a) A schematic showing the *mpsA* locus with *mspA* shown in dark grey and neighboring genes shown in light grey. **(b)** The toxicities of transposon mutants of *mpsA*-neighboring genes in both JE2 and SH1000 backgrounds were tested. NE42 showed a reduced toxicity compared to JE2, but a higher toxicity compared to JE2*mspA*::tn. In the SH1000 background, toxicities of these transposon mutants were similar to SH1000 wild type. (c) PSM abundance is not affect by the inactivation of gene to either side of *mspA*. TCA precipitated supernatants of the transposon mutants in both the JE2 and SH1000 background were separated on an SDS-PAGE gel. The PSM band runs ahead of the dye front as indicated.
